# Towards Patient-Oriented Diabetes Care: Results from Two KORA Surveys in Southern Germany

**DOI:** 10.1155/2015/368570

**Published:** 2015-03-17

**Authors:** Michaela Schunk, Renée Stark, Peter Reitmeir, Christa Meisinger, Rolf Holle

**Affiliations:** ^1^Institute of Health Economics and Health Care Management, Helmholtz Zentrum Munich, 85764 Neuherberg, Germany; ^2^Institute of Epidemiology II, Helmholtz Zentrum Munich, 85764 Neuherberg, Germany; ^3^German Center for Diabetes Research (DZD e.V.), 85764 Neuherberg, Germany

## Abstract

*Objective*. This study aims to examine the relationship of diabetes care processes and patient outcomes with an expanded set of indicators regarding patient-oriented care delivery, such as treatment satisfaction, the quality of patient-physician relationship, and a wider range of patient outcomes such as self-management, health behaviour, disease-related burden, and health-related quality of life (HRQL). *Methods*. The study population consisted of 486 participants with type 2 diabetes in two population-based follow-up surveys, conducted in 2003 to 2005 and 2006 to 2008 in Southern Germany. Data were self-reported and questionnaire-based, including the SF-12 for HRQL. Multiple regression models were used to identify associations between care processes and outcomes with adjustment for confounders. *Results*. Frequent medical examinations increased the likelihood of self-monitoring activities, such as foot care. A positive patient experienced relationship with their physician is associated with higher adherence to medical recommendations, such as medication intake, and the score of the SF-12 mental component. Participants with diabetes-related complications reported higher levels of medical examinations and multiprofessional care. *Conclusions*. Indicators of patient-oriented care should become an indispensable part of diabetes clinical practice guidelines with the aim of striving for more effective support of patients.

## 1. Introduction

Type 2 diabetes mellitus (T2DM) is a major global chronic disease with immense and increasing healthcare costs and a high disease burden. Complications are the main driver of diabetes care costs [1, 2]. Diabetes and diabetes-related complications considerably decrease health-related quality of life (HRQL) [3]. There is an indispensable need to study how diabetes care can further be improved to limit the disease burden and costs.

Health care systems attempt to deal with chronic diseases by standardizing treatment and clinical practice. Important policy strategies include the development of national clinical practice guidelines and indicators for the quality of care [4–7]. A review of observational studies examining the relationship between guideline-defined diabetes processes and health outcomes notes that effects are often surprisingly small and inconsistent [8].

Health care planners are optimistic about the capacity of patients to take responsibility for their health and the management of their chronic illness [9, 10]. The promotion of patients' involvement in the management of their chronic disease has become a popular concept and is delineated in the chronic care model and the patient empowerment approach [11, 12]. A patient-centered approach to diabetes care, endorsed as a central aim by the American Diabetes Association and the European Association for the Study of Diabetes [13], includes patient education but goes beyond this to embrace multiprofessional care, care management activities, and a proactive, respectful communication with patients. Glasgow et al. and others argue for a broader inclusion of patient-centered measures in studies of quality of diabetes care [14–16].

Studies have shown that diabetes care provided along these lines enhances metabolic control and clinical outcomes, in particular, when patients engage actively in their self-management [17–20]. Accordingly, we would argue that care delivery based on a partnership model between health professionals and patients should improve patient commitment to their health behaviour, self-management, and adherence, which can be conceptualised as patient outcomes.

Striving towards the use of patient-oriented quality of care indicators, the main limitation is a lack of empirical data [21]. Few clinical trials include patient-oriented indicators as primary outcomes [22]. In fact, clinical trials may not be very suitable for determining the impact of these indicators when implemented in actual practice. Population-based observational studies can offer both a view close to health care provision in practice and data on a wide range of structure, process, and outcome indicators of diabetes care.

Drawing on data from two population-based survey studies that were conducted in a region in Southern Germany from 2003 to 2008 we performed a post hoc analysis to test whether patients well supported by health professionals (as indicated by higher levels of care processes such as medical examinations, diabetes education, and multiprofessional care as well as high treatment satisfaction and good quality of the relationship between patients and physicians) attain higher levels of health behaviour and self-management and better adherence as well as less disease-related burden and higher HRQL.

## 2. Methods

### 2.1. Research Design

The study population consisted of 486 participants with T2DM who took part in either of two follow-up survey studies conducted in 2003–05 (F3) and 2006–08 (F4). F3 was a follow-up cohort study (*N* = 3184) to a 1994-1995 population-based MONICA survey (S3) with a response rate of 76%. F4 was a follow-up cohort study (*N* = 3080) of the KORA S4 survey conducted in 1999–2001 with a response rate of 80%. The KORA research platform (Cooperative Research in the Region of Augsburg) is a population-based data pool established in 1996 to continue and expand the MONICA project in Augsburg, a region with a mixed urban and rural population of about 600,000 inhabitants in the southern part of Germany, about 70 km west of Munich. Four cross-sectional health surveys (S1 to S4) have been performed in the city of Augsburg and its two surrounding counties at five-year intervals from 1984/85 onwards, each drawn as an independent random sample. The four cross-sectional surveys serve as cohorts for long term follow-up studies like F3 and F4. Both studies utilize the sampling in the original surveys, although data may not be fully representative due to death and loss to follow-up. Study designs, sampling methods, and data collection have been described in detail elsewhere [23].

Both surveys included a personal interview, several self-administered questionnaires (including the SF-12 questionnaire on HRQL), medical examinations, physical measurements, computer assisted documentation of medication during the last 7 days (IDOM), and laboratory tests. Data were collected at the study centre in Augsburg. The analysed data is based mainly on items from a questionnaire filled in by participants with diabetes but includes information collected in the interview (social-demographic data; type and duration of diabetes; cardiovascular comorbidity, BMI, physical activity, smoking, and alcohol) and SF-12 questionnaire data. IDOM data were used for the classification of diabetes treatment. Data quality was thoroughly checked in both studies. For the present analysis, only questions with identical wording and answer choice order were included to ascertain the validity of the joint analysis of both studies. Both studies were approved by the Ethics Committee of the Bavarian Medical Association and by the Bavarian Commissioner for Data Protection and Privacy. All study participants provided written consent.

### 2.2. Participants

Only study participants with type 2 diabetes in F3 and F4 were included in our analysis. Diabetes status and type of diabetes were assessed by a question in the interview (self-report of a physician-confirmed diagnosis of diabetes). Negative self-reports were checked with documentation of respective antidiabetes medication (ATC Codes: A10A and A10B) and corrected, if necessary. In case of doubt, the primary physician was contacted to confirm the diagnosis. Of the *n* = 3184 participants of F3, *n* = 259 were classified as participants with type 2 diabetes, respectively, *n* = 227 of the *n* = 3080 participants of F4. We excluded *n* = 11 (F3) respective *n* = 8 (F4) participants with self-reported type 1 diabetes to improve sample homogeneity. The sample for analysis consists of 486 participants in total, combining the data of those two surveys to increase sample size for the analysis. Participants over 75 years of age filled in a shortened version of the SF-12 in the F3 survey, leading to a higher number of missing values for HRQL.

### 2.3. Measurements

#### 2.3.1. Process Parameters

Diabetes care processes are defined in this study as elements of diabetes care that result from direct interactions between health professionals and patients. Processes of diabetes care were assessed with the following variables: (1) medical examinations and advice, (2) diabetes education, (3) treatment satisfaction, (4) patient-perceived quality of patient/physician relationship, and (5) multiprofessional care. Five types of medical examinations (eye exam, foot exam, HbA1c lab, blood pressure, and protein/urine testing) and two types of advice given by the physician on diet and physical exercise were assessed by the percentage of participants who reported this activity as having been performed during the last 12 months (yes/no). Diabetes education was assessed by the number of courses participants reported to have attended. Treatment satisfaction was assessed with a single item: “In total, how satisfied are you with the diabetes treatment (including treatment with insulin, tablets and/or diet) you have been receiving in the past weeks?” Responses from 1 (very dissatisfied) to 7 (very satisfied) were analyzed across the entire range. The quality of the patient/physician relationship was assessed by items relating to four different quality aspects: comprehensibility of information, opportunity to ask questions, shared decision-making, and psychological support. Responses for these variables were dichotomized as “excellent, good” versus “other” to ascertain good discriminatory power. Multiprofessional care was assessed by two items asking whether dieticians and/or podiatrists were involved in the diabetes care (yes/no) during the past 12 months.

To prepare process parameters for the multivariate analysis, we added up answers to respective questions in each group (medical examinations (7 items), quality of patient/physician relationship (4 items), and multiprofessional care (2 items)), which were identical in wording and answer format (see Table 5). Diabetes education (number of classes attended) and treatment satisfaction (range 1–7) were already available as single values.

Missing values were set to “0” except for missings with regard to treatment satisfaction, where the sample mean value of 5 was imputed. We excluded participants with >3 missings in the variables relating to process parameters to minimize the need for such imputations in this dataset with its many missing values (see Table 5) but to retain a workable sample size for the multivariate analyses.

#### 2.3.2. Outcome Parameters

In this study we perceive self-management, adherence, and health behaviour as intermediate patient outcomes, that is, activities that may result from care processes providing effective support of patients. Complications and health-related quality of life are considered as medium and long term outcome indicators. Taken together, patient outcomes were assessed with the following variables: (1) patient self-management, (2) adherence, (3) health behaviour, (4) diabetes complications, and (5) health-related quality of life.

Patient self-management was assessed with items regarding self-monitoring activities. Participants were asked whether and how frequently they had self-monitored feet, weight, blood glucose, and blood pressure within the past 6 months. Each item was dichotomized into “1” for frequencies of once a month or more and “0” for less. These activities are included in a validated scale of diabetes self-care activities (Summary of Diabetes Self Care Activities, SDSCA [24]), although this scale uses a different design to assess frequency.

Adherence was assessed with self-reported information on whether participants were following medical advice regarding medication, physical activity, diet, and foot care (i) without difficulties, (ii) with some difficulties, or (iii) with major difficulties. Each item was dichotomized into “1” for a report of no difficulties and “0” for some or major difficulties. For descriptive analysis, we also compared how many participants stated that they had not received recommendations. The respective answer category reads “does not apply to me” (Table 5).

For health behaviour, we used self-reported information on physical activity, smoking, and alcohol consumption and coded them in accordance with guidelines [25–27]. Physical activity is dichotomized as “1” (≥2 hr/week regularly in winter and summer) versus “0” (<2 hr/week regularly in winter, summer, or both). Smoking is dichotomized into “1” (never-smokers and ex-smokers) and “0” (regular and occasional smokers). Alcohol intake is transformed by using cut-off values of weekly consumption in gram (“0” = men > 20 g, women > 10 g; “1” = equal or less) [28].

Diabetes-related burden was assessed with regard to common diabetes complications and the occurrence of hyper- and hypoglycaemia. Diabetic complications refer to the clinical diagnosis (“ever” versus “never”) of retinopathy, poor blood circulation in legs, peripheral neuropathy, and microalbuminuria. The answer “do not know” is treated as missing value. Two items ask to recall the occurrence of hypoglycemic and/or hyperglycemic episodes during the past 6 months (yes versus no). HRQL is assessed with the SF-12, a validated questionnaire and short form of the SF-36 [29]. Values are expressed as two scores, the physical health score and mental health score (PCS and MCS). Both scores range from 0 to 100, with higher values indicating better HRQL. Scores are calculated using published standard algorithms [30].

All of the above variables are described in Table 5 with regard to their wording, the number of missing values, and the coding used for all analyses. Covariates included in the regression analysis are time of survey, patient age, sex, educational level (basic education versus higher), diabetes duration, diabetes treatment type, and comorbidities (history of stroke/myocardial infarction: yes/no). Treatment type refers to (1) no antidiabetic medication, (2) oral hypoglycemic agents (OHA), and (3) insulin treatment. Age and duration of diabetes are entered as continuous variables. For sample description we added information on the BMI (body mass in kilograms divided by the square of height in units of meters (kg/m2)), health insurance status (% statutory), and enrolment in a diabetes type 2 disease management program (T2DM-DMP).

### 2.4. Statistical Analysis

Descriptive statistics (percentages, means (SD)) were generated for sample characteristics and process and outcome parameters. Sample characteristics are reported in total and by treatment type. Differences between the populations of the two survey periods with time of survey entered as dichotomous variables (F3, F4) were tested by Student's* t*-test for continuous variables and *χ* ^2^-test for categorical variables. Multiple logistic regression models were used to examine the association between care processes with patient outcomes, controlling for age, sex, education, treatment type, cardiovascular comorbidities (previous myocardial infarction or stroke), and time of survey participation as covariates.

Odds ratios (OR) were calculated for binary variables. For continuous variables, such as quality of life scores, adjusted mean differences were based on a linear regression model.* P* values less than 0.05 are considered as statistically significant but used in an explanatory sense to retrieve further hypotheses. Statistical analysis was performed using SAS version 9.2 [31].

## 3. Results

### 3.1. Sample Description

The sociodemographic characteristics of the total sample and of groups divided by treatment type, (1) no antidiabetic medication, (2) oral hypoglycemic agents (OHA), and (3) insulin treatment, are shown in Table 1. About half of the sample was treated with oral hypoglycemic agents (OHA) (*n* = 259, 53%), with the remainder equally divided between those on insulin only and insulin combination treatment (*N* = 111, 23%) and those without antidiabetic medication (*N* = 116, 24%). The average age is 67.3 years and the proportion of female participants 44%. Average diabetes duration varies across the groups with different treatment types from 5.6 years in the group without antidiabetic medication to 16.2 years in the group with insulin treatment. Cardiovascular comorbidity and BMI were highest in the group with insulin treatment. Overall T2DM disease management-enrolment was 20%; it was lowest in the group with no medication. There were no differences between the survey periods regarding baseline variables entered in the regression analyses (Table 4).

### 3.2. Description of Process and Outcome Parameter

Table 2 shows the frequencies of care processes and patient outcomes across the sample. Blood pressure testing was the most frequent (95%) and feet exams the least frequent (50%) of medical examinations and advice. HbA1c testing at least once in the past 12 months was reported by 63%. Of the seven types of medical examinations and advice, participants received a mean of 4.2 (2.2 SD). Half of the participants had attended at least one patient education class (51%). Across all participants, the average participation completed one diabetes education class (SD 1.8). The mean value for treatment satisfaction was 5.3 (1.6 SD) on a rating scale from 1 (very dissatisfied) to 7 (very satisfied).

Quality of patient/physician relationship was rated high across all items. Only psychological support was acknowledged less often as being “good” or “excellent” (78%), compared with the comprehensibility of information (87%), the opportunity to ask questions (90%), and shared decision-making (85%). Of the four items, participants rated an average of 2.9 (SD 1.5) items as “good” or “excellent.” Participants reported low use of multiprofessional care: only 24% had used a podiatrist during the last 12 months, 17% a dietician.

Patient self-management, that is, monthly or more self-monitoring of blood-pressure, weight, feet, and blood-glucose, was reported by 65–80% of participants with the highest percentage for monitoring weight (81%). Differences between high adherence with respect to medication and feet care (>80%) and much lower adherence with regard to physical activity and diet (ca. 40%) were identified. Up to a quarter of participants stated that they did not receive respective medical recommendations. Only 14% of participants reported to engage in regular physical activity of more than 2 hours per week. However, more than 88% reported not smoking and 73% moderate/no alcohol consumption.

Disease-related burden was increased for 9% of participants with diabetic retinopathy, 12% with microalbuminuria, 21% with poor circulation in legs, and 29% with peripheral neuropathy. More than half of the participants stated to have experienced episodes of hyper- and hypoglycemia in the last 6 months. Mean HRQL was 41.5 in the physical component score (PCS-12) and 49.8 in the mental component score (MCS-12).

### 3.3. Evaluation of Relationship between Process and Outcome Parameters

Results of the regression analyses are presented in Table 3. We excluded *N* = 70 participants (14% of the total sample) because of >3 missing values in variables pertaining to care processes (F3: *N* = 49; F4: *N* = 21). Most of them were participants who did not fill in the diabetes-specific questionnaire (F3: *N* = 34 of *N* = 259; F4: *N* = 11 of *N* = 227).

Associations with patient self-management, health behaviour, disease-related burden, and health-related quality of life were strongest for medical examinations and advice, diabetes education, and quality of relationship with physician and less strong for treatment satisfaction and multiprofessional care. A higher number of medical examinations and advice were associated with self-monitoring of blood pressure (OR 1.18; CI: 1.03, 1.35), feet (OR 1.24; CI: 1.09, 1.42), and blood glucose (OR 1.23; CI: 1.07, 1.42). However, participants with more medical examinations and advice also reported less frequently that they had no difficulties with adherence to diet (OR 0.82; CI 0.70; 0.95). Participation in diabetes education was positively associated with self-monitoring feet (OR 1.30; CI 1.06, 1.60) and blood glucose (OR 1.50; CI 1.18, 1.90). Patient-perceived positive quality of relationship with the physician was most strongly associated with adherence to medication (OR 1.92; CI: 1.39, 2.64) and the mental component score of HRQL (mean score difference 2.04 CI: 0.95, 3.13). It was less strongly, but still significantly, associated with adherence to recommendations regarding diet (OR 1.33; CI: 1.04, 1.71) and feet care (OR 1.33; CI: 1.02; 1.73) as well as self-monitoring of weight (OR 1.26, CI: 1.00; 1.58). Multiprofessional care was positively associated with self-monitoring of blood glucose (OR 1.63, CI: 1.02, 2.62).

Contrary to our hypothesis, a number of associations indicated that participants reporting a higher level of care processes were more likely to report diabetes-related complications, episodes of hyperglycemia, and lower scores in the mental component of HRQL. Participants who receive multiprofessional care are less likely to be free of poor blood circulation in legs (OR 0.54; CI: 0.35, 0.83), peripheral neuropathy (OR 0.68; CI: 0.47, 0.99), and hyperglycemia (OR 0.61; CI: 0.40; 0.91). The risk of no retinopathy decreases with more diabetes education (OR 0.75; CI: 0.63; 0.90), and the physical component HRQL score is lower (mean score difference: −1.01; CI: −1.76; −0.26). The risk of no microalbuminuria decreases with a higher mean score in medical examination and advice (OR 0.79; CI: 0.63; 0.99). Finally, the only association found for treatment satisfaction indicates that participants with more treatment satisfaction are less likely to not have been diagnosed with retinopathy (OR 0.71; CI: 0.51; 0.98).

To investigate the robustness of our results, sensitivity analyses were performed. Models were calculated when (1) adjusting for diabetes duration instead of treatment type, (2) diabetes treatment in four (insulin only; insulin and OHA; OHA; no medication) versus three groups (insulin only or insulin and OHA; OHA; no medication), and (3) controlling for T2DM-DMP enrolment. In each case, the results proved to be very comparable (results not shown).

## 4. Discussion and Conclusion

### 4.1. Discussion

Measuring quality of care requires process and outcome measures that go beyond specific clinical interventions to integrate components that are guided by the patients themselves [14–16, 32]. We have tried to capture quality of care broadly by relating care processes to patient outcomes, expanding the set of indicators to include patients' activities and experiences. Controlling for treatment type, we aimed at results applicable across all patient groups. Our findings confirm a close relationship between care processes and patient outcomes. Following our hypotheses, frequent medical examinations increase the likelihood of self-monitoring activities. Patient-experienced positive relationship with physician is associated with higher adherence to medical recommendations, for example, medication and the SF-12 mental component score. Contrary to our expectation, higher levels of medical examinations and multiprofessional care did not preclude disease-related burden but were associated with it. This may indicate that patients' support by health professionals is being initiated too late and is only intensified once complications are present. This study, however, used a cross-sectional design, which introduces problems with confounding. We have adjusted for cardiovascular comorbidity and treatment type, but these adjustments may not be enough to control for the intensified health care needs of sicker patients (i.e., those with more complications). Further research with prospective studies is needed to reevaluate these findings.

On a descriptive level, our results provide evidence that the frequency of care processes still falls short in Germany. With the exception of blood pressure, all medical examinations required to be performed yearly are reported only from 50%–75% of participants. For example, annual eye exams are recommended for all patients with T2DM, but in our data only about two thirds of the patients reported receiving one in the past 12 months. Only 50–60% of participants reported receiving advice on diet or on physical exercise or ever participating in patient diabetes education. Similar findings have been reported by other studies using insurance claim data [33]. Guidelines recommend that all patients should receive these types of examinations and advice as well as patient education [7].

Patient self-monitoring levels as reported in this study must also be regarded as relatively low level, considering that 20–30% of patients do not carry out these activities at least on a monthly basis. However, in the light of diabetes treatment guidelines which only very generally recommend that physicians should review health behaviour with each patient on a regular basis (i.e., at least annually) this may be explainable [7, 34]. Although self-monitoring of weight was reported relatively more often compared to the other types of self-monitoring, adherence to medical recommendations concerning physical activities and diet was difficult for the majority of participants. Whilst adherence to most types of medical recommendations, except recommendations concerning physical activity, is higher when patients perceive the quality of patient-physician relationship to be good, patients' self-monitoring is associated with more frequent medical examinations, advice, and diabetes education. In the guidelines, self-monitoring of blood glucose should be encouraged by physicians as deemed suitable for the individual treatment. Reimbursement of glucose test strips has been cut for OHA-treated patients with diabetes in 2011 by the federal regulatory bodies [35]. They were however available at the time of the study.

Care processes were not associated with any of the three types of health behaviour which we assessed, that is, physical activity, smoking, and alcohol consumption. Only about 15% of participants report regular physical exercise. Participants do better managing smoking and alcohol consumption, but approximately 30% report a higher than recommended intake of alcohol and approximately 10% are smoking. These figures are consistent with data from a nationwide German health survey, carried out between 2008 and 2011 [36]. Patient education must address these issues more thoroughly and substantially increase its reach. Only half of the participants in our study state that they have attended diabetes education classes at least once. Loveman et al. (2008) conclude that patient education must have a clear program at the outset and be reinforced at additional points of contact and should be delivered by a team of educators [37]. There need to be more efforts to monitor age, sex, and socioeconomic differences in health behaviour to target interventions and evaluate these kinds of complex interventions.

In our study, we found that a higher patient-perceived quality of patient-physician relationship is associated with a higher score for the mental component of health-related quality of life (MCS-12), roughly equalling the difference in MCS-12 scores between women with and without diabetes [38]. This indicates the potential benefits of intensified patient-oriented care processes for patient outcomes.

The strength of this study is its population-based approach, providing data regardless of contact to medical care providers or membership in a particular sickness insurance fund. Baseline characteristics of our sample, such as the distribution across the treatment groups (no medication, OHA, and insulin), are similar to what has been found for patients with T2DM in other study samples in Germany [39]. We have used multiple logistic regression models and adjusted for important covariables such as education, cardiovascular comorbidity, and duration of diabetes. Models proved to be very robust when sensitivity analyses were performed, that is, when adjusting for diabetes duration instead of treatment type.

The questionnaire used in this study did not contain validated scales for the assessment of care processes or self-management, such as the Patient Assessment of Chronic Illness Care (PACIC) [40], Diabetes Management Self-Efficacy Scale (DMSES) [41], Diabetes Treatment Satisfaction Questionnaire (DTSQ) [42], or SDSCA scale, most of which are not available in German or have only recently been translated [24, 43]. We combined the items on medical examinations, quality of patient-physician relationship, and multiprofessional care to reduce complexity of the analysis. This must be regarded as exploratory and calls for further validation or the use of validated scales in future studies. With regard to measuring HRQL, the use of disease-specific quality of life questionnaires for type 2 diabetes in addition to generic questionnaires such as the SF-12 is important to capture the full spectrum of experiences with diabetes, including the psychological burden of the disease [44].

Some items had a large number of missing values. Thus the analysis was run on a smaller sample, removing participants with >3 missing values in process or outcome variables. The assessment of comorbidities and diabetic complications based on patient-reports may be particularly susceptible to information and recall bias. However, studies that use insurance data or review physicians' charts have found comparable rates of comorbidities and diabetic complications in Germany [45, 46]. Recall of medical examinations can also be biased. A comparison with data from the largest statutory health insurance fund in Germany shows good agreement with regard to HbA1c testing (in our data 64%, health insurance data 69%) but a higher frequency of self-reported eye exams (in our data 69%, health insurance data 35%) [33]. Other types of medical examinations studied here are not accounted for in administrative data in way that would be comparable to our data.

Our data only captured some aspects of quality of care while others may be added by other studies. Also, classifying the elements as done in this study is one way to conceptualise possible associations and there may be others which are equally valuable. For example, treatment satisfaction can be regarded as patient outcome, when factors not under control of the health care system or the health professionals are considered equally important. Following this, self-monitoring, adherence, and health behaviours can be treated as care processes when focusing on the interaction between health professional and patient to initiate, adopt, and maintain actions. In this study however, we perceive self-management, adherence, and health behaviour as intermediate outcomes, that is, activities that may develop when care processes work well beforehand.

Overall, our analysis should be regarded as explorative and qualitative in its approach. Generalizations from this rather small regional study with mainly self-reported data should be done carefully and only in the light of additional data. This study relied mainly on self-reported questionnaire data with its limitations in validity and reliability. However, the data offer rich insights on patients' perceptions of quality of diabetes care, which are rare and valuable for expanding our understanding of patient-oriented processes and outcomes in diabetes care.

### 4.2. Conclusion

Efforts to improve diabetes care need to go beyond guidelines to standardizing treatment and clinical practice and integrate indicators pertaining to higher levels of patient-oriented care processes and outcomes. Our study underlines the importance of monitoring and evaluating diabetes care by drawing on patient-reported indicators for both processes and outcomes as an indispensable part of clinical practice.

Our results stress the importance of finding more effective strategies to support patients to change health behaviour, in particular with regard to physical activity. Attention should be paid to fostering the patient-perceived quality of patient-physician relationship. Diabetes education must broaden its reach and scope, for example, in the field of health behaviour. Rather than programs delivered just once per patient, it must be remodelled into providing long-term support to maintain patient engagement [47, 48]. Further research is warranted to consider how diabetes self-management is associated with patients' prioritization of health outcomes and quality of life, caregiver support as well as costs.

## Figures and Tables

**Table 1:** Baseline characteristics of study population by treatment group.

	Total (*N* = 486)	No medication (*N* = 116)	OHA^a^ only (*N* = 259)	Insulin only or insulin with OHA (*N* = 111)^b^
Women (%)	43.8	45.7	40.5	49.6
Age (mean yrs (SD))	67.3 (9.2)	67.0 (9.5)	67.1 (9.1)	68.2 (9.0)
Duration of diabetes (mean yrs; (SD))	8.7 (8.4)	5.6 (6.8)	6.9 (6.6)	16.2 (9.4)
% in categories				
0–4 yrs	39.7	60.3	43.2	9.9
5–9 yrs	26.3	22.4	31.7	18.0
10–19 yrs	22.0	12.1	20.1	36.9
20–29 yrs	8.2	2.6	3.1	26.1
≥30 yrs	3.7	2.6	1.9	9.0
Education (% basic)	73.5	67.2	74.9	76.6
Cardiovascular comorbidity (%)^c^	17.9	14.7	13.1	32.4
BMI (kg/m^2^; mean (SD))	31.2 (5.1)	29.6 (4.4)	31.4 (4.9)	32.6 (5.8)
Health insurance (% statutory)	89.5	89.7	88.8	91.0
Enrolment in T2DM DMP (%)^d^	20.2	9.5	24.3	21.6

^a^OHA = oral hypoglycemic agents; ^b^only insulin 50.4%; insulin and OHA 49.6%.

^c^Previous myocardial infarction or stroke; ^d^Self-reported enrolment in structured disease management programs (T2DM DMP). These programs were not widely offered at the time of the F3 survey.

**Table 2:** Descriptive statistics.

		Percentage of total (*N* = 486)^a^

Care processes	Medical examinations (>once/last 12 months)	
	Eye exam	68.7
	Feet exam	50.3
	HbA1c testing	62.5
	Blood pressure	95.3
	Protein/urine testing	74.8
	Dietary advice	57.9
	Advice on physical exercise	56.0
	Frequency of medical exams/advice^b^ (0,7)^c^	4.2 (2.2)^d^
	Diabetes education (ever)	51.4
	Number of education classes (0,15)^c^	1.0 (1.8)^d^
	Treatment satisfaction (1,7) (last weeks)	5.3 (1.6)^d^
	Quality of patient/physician relationship (past 12 months)	
	Comprehensibility of information	87.2
	Opportunity to ask questions	90.0
	Shared decision-making	84.7
	Psychological support	77.7
	Frequency of positive ratings^b^ (0,4)^c^	2.9 (1.5)^d^
	Multiprofessional care (past 12 months)	
	Dietician	16.6
	Podiatrist	24.0
	Frequency of multiprofessional care input^b^ (0,2)^c^	0.3 (0.6)^d^

Patient outcomes	Self-monitoring (monthly or more %)	
	Blood pressure	73.0
	Weight	80.6
	Feet	69.8
	Blood glucose	64.6
	Patients' adherence to physician's recommendations^e^	
	Medication	90.0 (18.2)^e^
	Physical activity	38.9 (28.2)^e^
	Diet	41.5 (18.1)^e^
	Feet care	81.6 (25.3)^e^
	Health behaviour	
	Physical activity ≥2 hour/week	14.0
	Nonsmoking	87.9
	Alcohol use p.d. (≤20 g men; ≤10 g women)	72.8
	Complications (% ever diagnosed)	
	Retinopathy	9.0
	Poor blood circulation in legs	21.4
	Peripheral neuropathy	29.3
	Microalbuminuria	12.2
	Adverse conditions associated with diabetes (last 6 months)	
	Hyperglycemia	60.8
	Hypoglycemia	51.1
	Health-related quality of life SF-12	
	Physical score (PCS-12)	41.5 (10.2)^d^
	Mental score (MCS-12)	49.8 (11.0)^d^

^a^missings are excluded;  ^b^summary variables used in multivariate analysis. Missings are set to “0” or imputed with mean value in the case of treatment satisfaction; ^c^range (min,max);^ d^mean (SD); ^e^percentage with no difficulties to adhere (percentage stating “does not apply to me”).

**Table 3:** Association between care processes and patient outcomes.

	Medical examinations and advice^a^	Diabetes education^a^	Treatment satisfaction^a^	Quality of relationship with physician^a^	Multiprofessional care^a^
	Odds ratio (95% CI) *P* value				
Self-monitoring					
Blood pressure	**1.18 (1.03; 1.35) 0.0192**	0.99 (0.86; 1.13) 0.8385	0.96 (0.83; 1.11) 0.6119	0.88 (0.69; 1.11) 0.2641	1.22 (0.79; 1.87) 0.3730
Weight	1.14 (0.98; 1.32) 0.0990	0.97 (0.84; 1.11) 0.6519	0.94 (0.80; 1.11) 0.4764	**1.26 (1.00; 1.58) 0.0477**	1.12 (0.76; 1.90) 0.4422
Feet	**1.24 (1.09; 1.42) 0.0016**	**1.30 (1.06; 1.60) 0.0104**	0.88 (0.76; 1.03) 0.1017	1.21 (0.97; 1.50) 0.0876	0.99 (0.65; 1.50) 0.9629
Blood glucose	**1.23 (1.07; 1.42) 0.0045**	**1.50 (1.18; 1.90) 0.0009**	0.98 (0.84; 1.14) 0.7632	1.12 (0.89; 1.41) 0.3215	**1.63 (1.02; 2.62) 0.0420**
Adherence to					
Medication	0.95 (0.75; 1.20) 0.6541	1.13 (0.86; 1.49) 0.3875	0.98 (0.74; 1.29) 0.8769	**1.92 (1.39; 2.64) ** **<0.0001**	0.80 (0.41; 1.56) 0.5064
Physical activity	0.97 (0.83; 1.14) 0.7392	1.02 (0.85; 1.22) 0.8448	1.15 (0.97; 1.36) 0.1081	1.08 (0.84; 1.40) 0.5423	1.24 (0.79; 1.94) 0.3584
Diet	**0.82 (0.70; 0.95) 0.0067**	1.03 (0.92; 1.16) 0.5960	1.13 (0.97; 1.31) 0.1092	**1.33 (1.04; 1.71) 0.0253**	1.01 (0.68; 1.51) 0.9589
Feet care	1.02 (0.85; 1.23) 0.8333	1.11 (0.91; 1.35) 0.2993	1.16 (0.96; 1.40) 0.1254	**1.33 (1.02; 1.73) 0.0340**	0.76 (0.46; 1.25) 0.2810
Health behaviour					
Physical activity	1.16 (0.97; 1.40) 0.1095	1.04 (0.89; 1.22) 0.6130	1.08 (0.90; 1.31) 0.4117	1.04 (0.76; 1.43) 0.7946	1.29 (0.79; 2.11) 0.3086
Nonsmoking	1.09 (0.90; 1.31) 0.3754	1.03 (0.82; 1.29) 0.8184	1.00 (0.82; 1.23) 0.9860	1.21 (0.91; 1.61) 0.1831	0.65 (0.38; 1.13) 0.1292
Moderate/no alcohol	0.97 (0.84; 1.11) 0.6254	1.15 (0.98; 1.36) 0.0931	0.96 (0.82; 1.12) 0.5818	0.98 (0.77; 1.25) 0.8859	0.84 (0.56; 1.27) 0.4070
Complications (none)					
Retinopathy	0.99 (0.77; 1.27) 0.9232	**0.75 (0.63; 0.90) 0.0015**	**0.71 (0.51; 0.98) 0.0387**	1.12 (0.73; 1.71) 0.6073	0.96 (0.49; 1.88) 0.9054
Poor blood circul. in legs	0.89 (0.75; 1.05) 0.1612	1.06 (0.92; 1.22) 0.4186	1.00 (0.85; 1.17) 0.9487	1.28 (0.99; 1.66) 0.0622	**0.54 (0.35; 0.83) 0.0053**
Peripheral neuropathy	0.98 (0.85; 1.12) 0.7322	1.00 (0.88; 1.14) 0.9927	1.05 (0.91; 1.21) 0.4844	1.13 (0.90; 1.41) 0.2872	**0.68 (0.47; 0.99) 0.0463**
Microalbuminuria	**0.79 (0.63; 0.99) 0.0386**	0.84 (0.68; 1.04) 0.1079	0.85 (0.66; 1.09) 0.2020	1.37 (0.96; 1.93) 0.0804	1.23 (0.67; 2.27) 0.5125
Adverse conditions (none)					
Hyperglycemia	1.13 (0.99; 1.29) 0.0753	1.00 (0.88; 1.14) 0.9629	1.09 (0.95; 1.26) 0.2152	1.03 (0.82; 1.28) 0.8277	**0.61 (0.40; 0.91) 0.0163**
Hypoglycemia	1.06 (0.93; 1.20) 0.3967	0.96 (0.85; 1.09) 0.5279	0.99 (0.86; 1.13) 0.8455	1.18 (0.95; 1.46) 0.1295	0.78 (0.53; 1.15) 0.2055

	Adj. mean differences (95% CI)				
Health-related quality of life					
(SF-12)					
Physical score (PCS-12)	0.001 (−0.63; 0.63) 0.9986	−0.60 (−1.29; 0.10) 0.0917	0.51 (−0.15; 1.16) 0.1287	1.00 (−0.01; 2.01) 0.0522	−0.86 (−2.66; 0.94) 0.3502
Mental score (MCS-12)	0.02 (−0.66; 0.70) 0.9513	−**1.01 (**−**1.76; **−**0.26) 0.0080**	0.17 (−0.54; 0.87) 0.6469	**2.04 (0.95; 3.13) 0.0002**	−0.89 (−2.83; 1.05) 0.3706

^a^ *N* = 416, excluding *N* = 70 with >3 missings in variables measuring care processes (see Table 2).

**Table 4:** Baseline characteristics of F3 and F4 study population.

	F3-Study 2004-2005 (*n* = 259)^a^	F4-Study 2006–2008 (*n* = 227)^a^	*P*-value
Women (%)	46.3	41.0	0.2345
Age (years, mean (SD))	66.9 (9.5)	67.9 (8.8)	0.2442
Duration of diabetes (in years; mean (SD))	9.0 (9.1)	8.4 (7.6)	0.3755
% in categories			0.3637
0–4 yrs	39.8	39.7	
5–9 yrs	23.6	29.5	
10–19 yrs	22.4	21.6	
20–29 yrs	9.7	6.6	
≥30 yrs	4.6	2.6	
Education (% basic)	73.4	73.6	0.9353
Cardiovascular comorbidity (%)^b^	18.9	16.7	0.5319
BMI (kg/m^2^, mean (SD))	31.2	31.3	0.6823
Type of diabetes treatment (%)	13.5	9.3	0.5341
Insulin	11.2	11.5	
Insulin and OHA	51.8	55.1	
OHA	23.6	24.2	
Diet			
Health insurance (% statutory)	87.3	92.1	0.0842
Enrollment in T2DM DMP (%)^c^	13.1	28.2	<0.0001

^a^Values are presented as percentages; ^b^MCI or stroke; ^c^Self-reported enrolment; T2DM-DMPs were not widely offered at the time of the F3 survey.

**Table 5:** Survey items.

Item	Wording	Number of missings F3/F4 (*N* = 259/227)	Coding
Care processes			
Medical examinations	The following list specifies medical examinations and advice which can be of importance in diabetes care. When was the last time your doctor did perform the following? (i) Eye exam (ii) Feet exam (iii) HbA1c testing (iv) Blood pressure (v) Proteinuria testing (i) Advice on appropriate physical activity (ii) Advice on appropriate diet	31/11 36/13 37/12 30/11 30/11 35/12 36/13	1 = in the last 12 months 2 = More than one year ago 3 = Never 4 = Do not know for multivariate analysis: if variable IN (1) then examination = 1; if variable IN (2, 3, 4) then examination = 0; for multivariate analysis: if answer is missing then examination = 0;

Diabetes education	Have you ever attended a diabetes education class? If yes, how many diabetes education classes have you attended?	38/16	1 = Yes 2 = No if variable IN (2) then education = 0; if variable IN (1) then education = number of classes for multivariate analysis: if answer is missing then education = 0; if variable IN (1) and number of classes IN (.) then number of classes = 1

Multiprofessional care	Have you been seen by any of the following health care professionals for your diabetes care during the past 12 months? (i) Podiatrist (ii) Dietician	51/18 58/18	1 = Yes 2 = No if variable IN (1) then interdisciplinary = 1; if variable IN (2) then interdisciplinary = 0; for multivariate analysis: if answer is missing then interdisciplinary = 0;

Quality of patient/physician relationship	The following questions refer to the performance of the physician who has been responsible for your diabetes care during the past 12 months: How do you rate (i) the comprehensibility of information which you receive? (ii) the opportunity to ask questions during your appointments? (iii) the opportunity to shared decision-making about your diabetes treatment? (iv) the psychological support provided from the physician?	45/20 45/20 47/21 51/23	1 = Bad 2 = Mixed 3 = Good 4 = Excellent if variable IN (3, 4) then quality = 1; if variable IN (1, 2) then quality = 0; for multivariate analysis: if answer is missing then quality = 0;

Treatment satisfaction	Overall, how satisfied are you with your diabetes treatment (including your therapy with insulin, tablets, and/or diet) during the last weeks?	63/30	Rating scale from 1 to 7 1 (very dissatisfied),…, 7 (very satisfied) for multivariate analysis: if answer is missing then treatment satisfaction = 5

Patient outcomes			
Self-monitoring	In the past 6 months: how often have you performed the following examinations at home? (i) check your weight? (ii) check your feet for wounds and skin cracks? (iii) measure your blood-pressure? (iv) measure your blood-glucose?	35/19 36/20 37/20 35/19	1 = daily 2 = once or several times per week 3 = 1–3 times per month 4 = less than once per month 5 = never if variable IN (1, 2, 3) then self-monitoring = 1; if variable IN (4, 5) then self-monitoring = 0;

Adherence	How difficult do you find it to stick exactly to the physician's recommendation concerning (i) medication intake? (ii) regular physical activity? (iii) keeping dietary advice? (iv) checking feet?	39/20 44/20 40/20 43/20	1 = very difficult 2 = somewhat difficult 3 = not difficult at all 4 = does not apply to me if variable IN (3) then adherence = 1; if variable IN (1, 2) then adherence = 0; if variable IN (4) then adherence =.;

Physical activity	How often do you engage in physical activity (in winter/summer)?	0/0	1 = regularly more than 2 hours p/week 2 = regularly approx. 1 hour p/week 3 = intermittently approx. 1 hour p/week 4 = hardly any or no physical activity if variable IN (1) then sports = 0; if variable IN (2, 3, 4) then sports = 1;

Smoking	Are you a regular smoker or smoking at convenience?	1/0	1 = regular smoker 2 = occasional smoker 3 = ex-smoker 4 = never smoked if variable IN (1, 2) then smoker = 1; if variable IN (3, 4) then smoker = 0;

Alcohol	Which alcoholic beverages did you consume during the past weekdays/weekend?	1/0	1 = no alcohol intake 2 = alcohol intake >0–<20 g/day 3 = alcohol intake >20–<40 g/day 4 = alcohol intake >40–<60 g/day 5 = alcohol intake >60–80 g/day 6 = alcohol intake more than 80 g/day alkkon = g/day if sex = male and variable IN (1, 2) then alcohol = 0; if sex = male and variable IN (3, 4, 5, 6) then alcohol = 1; if sex = female and alkkon =<10 then alcohol = 0; if sex = female and alkkon >10 then alcohol = 1;

Complications	This is a list of diseases of the eye. Have you ever been diagnosed with any of them by a physician? (i) Diabetic retinopathy (diseases that affect the macula of the eye). Has a physician diagnosed you ever with any of the following diseases of the kidney or the bladder? (i) Microalbuminuria Has a physician diagnosed you ever with any of the following diseases of the feet or legs? (i) Poor blood circulation in legs (ii) Peripheral neuropathy (numbness, Burning, and prickling)	49/16 47/11 41/11 38/11	1 = Yes 2 = No 3 = Do not know if variable IN (1) then complication = 1; if variable IN (2) then complication = 0; if variable IN (3) complication is coded as missing

Conditions associated with diabetes	How often during the last 6 months have you felt signs of high or low blood sugar? (i) Signs of high blood sugar (e.g. thirst, frequent passing of water) (ii) Signs of low blood sugar (e.g. sweating, faintness, trembling)	52/13 48/17	1 = once or several times per week 2 = 1–3 times per month 3 = less than once per month 4 = never if variable IN (1, 2, 3) then Condition = 1; if variable IN (4) then Condition = 0;

Health-related quality of life	SF-12 items (time frame of items: last 4 weeks)	111^*^/13	Calculation of summary scores according to Bullinger and Kirchberger (1998) [30]

^*^In the F3 survey, SF-12 data were not collected for respondents ≥75 years.

## Authors' Declaration

I confirm all patient/personal identifiers have been removed or disguised so the patient/person(s) described are not identifiable and cannot be identified through the details of the story.
